# Knowledge and practice of induction of lactation in trans women among professionals working in trans health

**DOI:** 10.1186/s13006-020-00308-6

**Published:** 2020-07-16

**Authors:** Emily Trautner, Megan McCool-Myers, Andrea Braden Joyner

**Affiliations:** 1 Department of Gynecology and Obstetrics, Emory University School of Medicine, Atlanta, GA USA; 2 Jane Fonda Center for Adolescent Reproductive Health, Emory University, Atlanta, GA USA

**Keywords:** Breastfeeding protocols, Lactation induction, Trans women, Transgender, LGBTQ+, LGBTQIA

## Abstract

**Background:**

Breastfeeding is emerging as an important reproductive rights issue in the care of trans and gender nonconforming people. This study sought to understand the tools available to professionals working in the field of trans health to help trans women induce lactation and explore the concept of unmet need.

**Methods:**

In November 2018, we conducted a cross-sectional study which surveyed attendees at the World Professional Association for Transgender Health (WPATH) symposium in Buenos Aires, Argentina. Eligible participants were 18 + years old, had professional experience with transgender populations, were able to complete a survey in English, and were conference attendees. Descriptive data were collected using a 14-item written survey encompassing demographic characteristics, experience in transgender health, and lactation induction in trans women.

**Results:**

We surveyed 82 respondents (response rate 10.5%), the majority of whom were healthcare professionals (84%). Average age of respondents was 42.3 years old. They represented 11 countries and averaged 8.8 years of work at 21.3 h/week with trans populations. Healthcare professionals in this sample primarily specialized in general/internal medicine, psychology, endocrinology, and obstetrics/gynecology. One-third of respondents (34%) stated that they have met trans women who expressed interest in inducing lactation. Seventeen respondents (21%) knew of providers, clinics, or programs that facilitated the induction of lactation through medication or other means. Seven respondents (9%) have helped trans women induce lactation with an average of 1.9 trans women in the previous year. Two protocols for lactation induction were mentioned in free text responses and 91% believe there is a need for specialized protocols for trans women.

**Conclusion:**

This exploratory study demonstrates healthcare professionals’ interest in breastfeeding protocols for lactation induction in trans women. Additional studies are needed to capture insights from breastfeeding specialists, e.g. lactation consultants and peripartum nurses, and to understand patients’ perspectives on this service.

## Background

As trans individuals are able to utilize gender-affirming treatments earlier in their adolescence, reproductive rights and access are emerging as significant issues in the care of trans and gender non-conforming people [1]. Breastfeeding is an important component of reproduction and provides a myriad of health and social benefits to both the breastfeeding individual and infant [2].

There has been some literature documenting the induction of lactation in cisgender women – individuals who were assigned female sex at birth and identify as a woman – in cases of adoption or surrogacy [3–7]. These protocols include the use of high estradiol and progesterone levels with later reduction to simulate birth, a galactagogue, and a breast pump for regular interval nipple stimulation [3–7].

Two qualitative studies documented the experience of inducing lactation in transmasculine and gender non-conforming persons who were assigned female sex at birth [8, 9]. Many individuals reported dysphoria with chestfeeding and the means of communication around the practice. Breastfeeding or chestfeeding in transmasculine patients can be achieved by a similar technique as inducing lactation in cis women after the discontinuation of testosterone therapy.

In January 2018 the first formal report of induction of lactation in a trans woman was documented in the medical literature [10]. The patient in the case report induced lactation with the aid of domperidone, estradiol, progesterone, spironolactone and regular nipple stimulation. A search of PubMed using the search terms “transgender” and “breastfeed*” or “lactat*” in May 2020 reveals no further entries addressing this specific clinical scenario. However, other grey literature sources report cases of induced lactation in trans women as early as 2010 [11, 12].

Transgender women and men seeking breastfeeding and chestfeeding support are a growing population [13]. However, it is unclear where trans patients are seeking support, and whether there are tools available to health professionals to help induce lactation. The objectives of this study were therefore to assess the demand for and awareness of existing protocols or methods for inducing lactation in trans women as reported by survey respondents.

## Methods

We conducted a cross-sectional study using a written survey to explore the reported demand and protocols for lactation induction in trans women. In July 2018, we performed a literature search of PubMed using combinations of the search terms “breastfeed*”, “lactat*”, “transgender”, and “survey” to identify validated surveys which could be used or adapted for this research question and setting. No relevant surveys were identified. The research team therefore drafted a survey with closed questions with a final open question asking for any other feedback. The survey sections captured demographic characteristics, including experience working with transgender populations, and the practice and knowledge of lactation induction. The drafted survey was developed in collaboration with an expert in survey development (MD, MPH) and a trans woman with a medical and analytical background (MD, PhD). The distributed survey included 14 items and can be found in Additional file 1.

Due to the paucity of literature, it is unclear where trans women seek support for lactation induction. Therefore, we decided to survey attendees at the World Professional Association for Transgender Health (WPATH) symposium in Buenos Aires in November 2018 with approval from conference organizers. WPATH is one of the largest trans studies conferences in the world, with 780 (Blaine Vella, personal communication, 15 May 2019) academic researchers, activists, and healthcare practitioners registered for the 2018 symposium [14]. We hypothesized that the clinical practice of lactation induction would fall within the scope of diverse health professions. However, professionals outside of clinical care would not be excluded from the study, due to the exploratory nature of this research. Therefore, the inclusion criteria were: 18 years of age or older, ability to complete a written survey in English, and registration at the symposium.

We disseminated surveys both passively and actively during the symposium, in paper format. Surveys were displayed on a table opposite the registration with posted information indicating that the survey was available to all symposium participants. We also placed surveys on seats in select lecture halls. Additionally, we actively distributed surveys before the start of lecture sessions specifically targeting clinical sessions in order to reach health professionals. Participants were permitted to fill out the survey during the session, or at their convenience, and return completed forms to the reception.

Once we collected all paper surveys, we performed dual data entry in an electronic spreadsheet. Reliability between subsequent entries was 99.3%. We corrected identified errors in data entry. Data were missing on several surveys, however since all surveys met a minimum of 80% completed, we included all results. If data were missing for a given variable, the denominator for assessment was adjusted to reflect the actual number of responses gathered. We performed descriptive data analyses with Microsoft Excel and SPSS Statistics Version 25. We used chi squared tests to measure the significance of association between variables. A *p* - value of < 0.05 was considered significant.

## Results

From November 3–6, 2018, we distributed our survey onsite to participants of the WPATH symposium. Eighty-two completed surveys were included in the analysis, yielding a response rate of 10.5% when factoring in the number of conference attendees (*n* = 780). For those individuals directly approached by the research team, reasons given for not participating in the survey were: they were not a clinical professional, they do not know anything about breastfeeding, and/or they were not interested. Table 1 presents the demographic characteristics of the respondents.

**Table 1:** Baseline demographic characteristics (*N* = 82)

Demographic	Mean ± SD / ***N*** (%)
Age in years	42.3 ± 9.9
Education level	
College	8 (10)
Masters	20 (24)
Doctorate	54 (66)
Gender identity	
Female	47 (57)
Male	22 (27)
Trans gender/queer/non-binary^a^	13 (16)
Profession^b^	
Physician	50 (61)
Therapist/Psychologist	11 (13)
Advanced care practitioner	7 (9)
Social worker	5 (6)
Public health researcher	4 (5)
Activist/community organizer	3 (4)
Nurse	1 (1)
Student	1 (1)
Other	5 (6)
Location of transgender work^c^	
United States	59 (72)
Canada	7 (9)
Europe	7 (9)
Oceania	6 (7)
Latin America and Puerto Rico	3 (4)
Africa	1 (1)
Work in a transgender clinic or health facility	65 (80)
Hours per week on transgender work	21.3 ± 16.6

^a^All respondents who reported a gender that was different than their sex assigned at birth were classified as transgender/queer/non-binary

^b^ Respondents could report more than one profession

^c^ Respondents could report more than one location of work

### Respondent demographic characteristics

Respondents had worked with transgender populations for 8.8 years on average (SD 6.4, range 1–32 years, median 7 years) and spent over half their work week contributing to the health needs and rights of transgender individuals and communities (mean 21.3 h, median 17.5 h, range 0–80 h). Respondents also reflected a variety of gender identities and 16% reported a non-cis-gender identity. Healthcare professionals outweighed non-healthcare professionals, with 69 respondents (84%) reporting a healthcare profession. Some health professionals provided more than one specialty, and Table 2 illustrates the primary specialties reported.

**Table 2:** Primary health specialties (*N* = 69)

Specialty	***N*** (%)
General Medicine/Internal Medicine	17 (25)
Psychology/Therapy/Psychiatry	15 (22)
Endocrinology	12 (17)
Pediatrics	6 (9)
Obstetrics/Gynecology	4 (6)
Lactation Consultant	2 (3)
Physiotherapy	2 (3)
Plastic surgery	2 (3)
Midwife	1 (1)
Dermatology	1 (1)
Hematology	1 (1)
Radiology	1 (1)
Urology	1 (1)
Health specialty not specified	4 (6)

### Demand for and provision of lactation induction services

One-third of respondents (*n* = 28, 34%) reported meeting trans women who expressed interest in inducing lactation. These respondents primarily worked in settings in the US and Canada (*n* = 25), while some worked in Oceania (*n* = 2) and Latin American (*n* = 1). Respondents were more often health professionals (*n* = 21 vs. *n* = 7, *p* = 0.098) and more likely to be employed at a transgender clinic or facility (*n* = 23 vs. *n* = 5, *p* = 0.000). Health professionals who had met women interested in inducing lactation primarily specialized in General Medicine/Internal Medicine (*n* = 8), Endocrinology (*n* = 4), Psychology/Therapy/Psychiatry (*n* = 4), and Obstetrics/Gynecology or Lactation Consulting (*n* = 3). The non-healthcare professionals reported careers in social work (*n* = 3), public health (*n =* 2), law (*n* = 1), and activism/advocacy work (*n* = 1).

Seventeen respondents (21%) knew of providers, clinics, or programs that helped trans women induce lactation. Seven respondents (9%) reported directly assisting trans women induce lactation or working in a clinic that provided this service. These respondents reported working in Canada (*n =* 2) or the US (*n* = 5). All were health professionals and specialized in the fields of General medicine/internal medicine (*n* = 3), Obstetrics/gynecology (*n* = 2), Endocrinology (*n* = 1), and Psychology/therapy/psychiatry (*n* = 1). Furthermore, respondents indicated that an average of 1.9 trans women were assisted to induce lactation in the previous 12 months at their sites (range 0–4). Three additional respondents indicated with a written comment that they would offer assistance if a trans woman wanted to induce lactation.

When prompted for any other feedback, several respondents indicated that access to a protocol and additional training would be helpful for their practice, since this was an infrequent request. One respondent reported that she worked with a 55-year-old trans woman who induced lactation using a protocol on social media and medication ordered online for the experience of lactation itself (with no intention to feed a baby). Respondents also reported the need for chestfeeding guidance for trans men and one respondent indicated that they were currently conducting a pregnancy and childbirth study in trans men that addresses chestfeeding.

### Induction of lactation protocols

In addition to experience with interest in induction of lactation, we asked respondents if a protocol for trans women was necessary. A large majority (*n* = 72, 91%) believe there is a need for specific breastfeeding protocols for trans women. Twenty-one participants (27%) were familiar with existing protocols. Relevant protocols named were listed as Mount Sinai/Zil Goldstein case study and Newman-Goldfarb [10, 15]. Other respondents wrote in web addresses for additional sources [11, 16]. In addition, other respondents reported knowledge of unpublished or grey literature methods and resources.

## Discussion

We found that survey participants represented diverse gender identities and countries; spent half their week working with transgender populations or issues; and a majority were in a clinical profession in a diverse set of specialties. A third reported they had met a trans woman who desired to induce lactation, and one in five reported knowledge of providers or clinics that were able to help trans women breastfeed. Less than one in ten reported that they or someone in their clinical practice had helped trans women induce lactation. Survey respondents overwhelmingly agreed (over 90%) that a breastfeeding protocol for trans women should be available for providers, however only one in four respondents reported current knowledge of an appropriate protocol. These data suggest that there is interest in lactation induction for both providers and patients.

Respondents reported two specific protocols, however there is no data to demonstrate efficacy or safety of either protocol. The 2018 case report of lactation induction in a trans woman at Mount Sinai was also referred to as the Zil Goldstein protocol [10]. Respondents also mentioned the Newman-Goldfarb protocol, which was initially designed for a cis woman to breastfeed a baby born to a surrogate mother [16]. The Mount Sinai case study starts with 10 mg domperidone three times daily while the Newman-Goldfarb protocol recommends 10 mg domperidone four times daily. Both regimens subsequently increase the dose to 20 mg four times daily. Since domperidone is not approved by the Food and Drug Administration [17], the patient in the Sinai case obtained the drug elsewhere. Both regimens utilize estradiol and progesterone, however the Mount Sinai protocol prescribes higher doses as compared to the minimum doses recommended by the Newman-Goldfarb protocol [10, 16]. Lastly, the Mount Sinai protocol employs spironolactone to prevent masculinizing effects of testosterone [10]. Informal reports of trans women who induced lactation have also emerged recently [12]. However, there are no data to support any of the other reports. Our survey findings, along with the peer-reviewed and grey literature, indicate a critical gap in evidence-based medicine for this growing patient population [13].

Although we used a number of approaches to recruit survey participants at the conference – displaying adjacent to the registration table, distributing at sessions, and distributing face-to-face to health care professionals – the response rate of our study was low (10.5%, 82/780). Over half of the surveys distributed to conference participants were returned completed (51.9%, 82/158). This corresponds with other paper-based, in-person response rates of 56–57% [18, 19]. Additional research staff to distribute the surveys, a post-conference email survey, and compensation for survey completion may have improved the overall response rate. Nonetheless, the participant demographic characteristics shed light on the medical specialties that report experience and/or interest in this health service. Other professionals who are responsible for breastfeeding support, such as lactation consultants and peripartum nurses, should be recruited for future studies.

## Conclusion

This exploratory study, while small in size, contributes to the body of peer-reviewed literature on reproductive health services for trans patients. The Academy of Breastfeeding Medicine (ABM) recently published a guide to lactation care for LGBTQ+ individuals, which is an important step towards providing appropriate care for this population [20]. In addition to clinical research on evidence-based breastfeeding protocols such as those outlined in the ABM guide, qualitative research is needed to understand patients’ perspectives on this health service. Literature to date has found that transgender women and families face considerable stigma and challenges in the process of breastfeeding and chestfeeding [13, 21]. These hurdles limit equal access to care for this patient population [21]. Future work should capture the knowledge, attitudes, and practices of these individuals and families with regard to breastfeeding to identify care priorities and barriers.

## Supplementary information

**Additional file 1.** Survey questions. This file contains the survey questions that were distributed to study participants.

## Data Availability

The datasets generated and analysed during the current study are not publicly available due to identifying information gathered from participants but deidentified data may be made available from the corresponding author on reasonable request.

## Abbreviations

LGBTQ+: Lesbian, gay, bisexual, transgender, queer, plus (other identities)
WPATH: World Professional Association for Transgender Health
