# Supplementary material for: Knowledge and practice of induction of lactation in trans women among professionals working in trans health
Source: Int Breastfeed J. 2020 Jul 16;15:63. doi: 10.1186/s13006-020-00308-6 (PMC7364529; doi:10.1186/s13006-020-00308-6)
Supplement: Supplementary file 1 — Additional file 1. Survey questions. This file contains the survey questions that were distributed to study participants. [file 13006_2020_308_MOESM1_ESM.docx]

**Additional file 1: Survey questions**

1. How old are you? ______

2. What is the highest level of education you completed?

[ ] Less than a high school diploma

[ ] High school diploma or GED

[ ] Some college, Associate’s Degree, or Technical Degree

[ ] College degree

[ ] Post graduate / Master’s degree

[ ] Doctoral degree

3. What was your sex assigned at birth?

[ ] Female

[ ] Intersex/ambiguous

[ ] Male

[ ] I prefer not to answer

[ ] Don't know

4. What best describes your current gender?

[ ] Female

[ ] Transgender

[ ] Male

[ ] I prefer not to answer

[ ] Other (please specify) *What is your other gender identity?*_______________

5. What best describes your profession?

[ ] Nurse

[ ] Social worker

[ ] Public health researcher

[ ] Activist/community organizer

[ ] Therapist

[ ] Physician (MD/DO)

[ ] Advanced care practitioner (i.e. nurse practitioner, physicians assistant)

[ ] Student – *Please specify field of study:*______________________________

[ ] Other:________________________________________________________

*If you work in the medical field, what is your most recent specialty or area of training?*

[ ] Psychiatry

[ ] Internal Medicine

[ ] Endocrinology

[ ] Family Medicine

[ ] Pediatrics

[ ] Plastic Surgery

[ ] Urology

[ ] Obstetrics and Gynecology

[ ] Other surgical specialty *Please specify:*_______________________________

[ ] Other:_________________________________________________________

6. How many hours on average per week do you spend working on matters related to the health and rights of transgender individuals and communities? _________________

7. How many years have you worked, volunteered or advocated for transgender populations? ___________________________________________________________

8. Do you work in a clinic or health facility that specifically addresses the needs of transgender patients?

[ ] No

[ ] Yes

*If yes please specify the clinic and the clinic location*:___________________________

9. In which countries, states or cities do you do most of your work with transgender individuals and communities?

10. Have you ever met any trans women who have expressed interest in inducing lactation?

[ ] No

[ ] Yes

*If yes, did you meet these trans women in a clinical setting or in a community network?*

[ ] Clinic setting

[ ] Community network

[ ] Other (please specify): __________________________________________________

*If in a clinical setting,* *how many trans women have expressed interest in breastfeeding induction in the past year within your clinical setting?____________________________*

11. Do you know of providers, clinics or programs that have facilitated the induction of lactation through medication or other means in trans women?

[ ] No

[ ] Yes

*If yes, approximately how many trans women received consultation about breastfeeding induction in the past year?*_________________________________________________

12. Do you, your clinical practice, or community network help trans women to induce lactation?

[ ] Not applicable

[ ] No

[ ] Yes

*If yes, how many trans women received consultation about breastfeeding induction in the past year?*___________________

*If yes, what is the name/contact information of the individual, clinical practice or network that provides these services?__________________________________________*

13. Do you know about specific methods or protocols used to induce lactation in trans women patients in informal practice settings (e.g. community networks)?

[ ] No

[ ] Yes

*If yes, please provide any additional information:*______________________________

14. Do you think that there is a need for a specific breastfeeding protocol for trans women patients?

[ ] No

[ ] Yes

Optional: Please provide any other information that you have about inducing lactation and breastfeeding in transgender populations in the space below.
